# Inhibitory Effects of Antiviral Drug Candidates on Canine Parvovirus in F81 cells

**DOI:** 10.3390/v11080742

**Published:** 2019-08-13

**Authors:** Hongzhuan Zhou, Xia Su, Lulu Lin, Jin Zhang, Qi Qi, Fangfang Guo, Fuzhou Xu, Bing Yang

**Affiliations:** Beijing Key Laboratory for Prevention and Control of Infectious Diseases in Livestock and Poultry, Institute of Animal Husbandry and Veterinary Medicine, Beijing Academy of Agriculture and Forestry Sciences, No. 9 Shuguang Garden Middle Road, Haidian District, Beijing 100097, China

**Keywords:** canine parvovirus, FDA-approved drug library, antiviral inhibitors, cytopathic effect (CPE)-based high-throughput screening assay

## Abstract

Canine parvovirus (CPV) is a common etiological agent of acute enteritis, which occurs globally in domestic and wild carnivores. Despite the widespread use of inactivated or live attenuated vaccines, the emergence of antigenic variants and the influence of maternal antibodies have raised some concerns regarding the efficacy of commercial vaccines. While no specific antiviral therapy for CPV infection exists, the only treatment option for the infection is supportive therapy based on symptoms. Thus, there is an urgent medical need to develop antiviral therapeutic options to reduce the burden of CPV-related disease. In this study, a cytopathic effect (CPE)-based high-throughput screening assay was used to screen CPV inhibitors from a Food and Drug Administration (FDA)-approved drug library. After two rounds of screening, seven out of 1430 screened drugs were found to have >50% CPE inhibition. Three drugs—Nitazoxanide, Closantel Sodium, and Closantel—with higher anti-CPV effects were further evaluated in F81 cells by absolute PCR quantification and indirect immunofluorescence assay (IFA). The inhibitory effects of all three drugs were dose-dependent. Time of addition assay indicated that the drugs inhibited the early processes of the CPV replication cycle, and the inhibition effects were relatively high within 2 h postinfection. Western blot assay also showed that the three drugs had broad-spectrum antiviral activity against different subspecies of three CPV variants. In addition, antiapoptotic effects were observed within 12 h in Nitazoxanide-treated F81 cells regardless of CPV infection, while Closantel Sodium- or Closantel-treated cells had no pro- or antiapoptotic effects. In conclusion, Nitazoxanide, Closantel Sodium, and Closantel can effectively inhibit different subspecies of CPV. Since the safety profiles of FDA-approved drugs have already been extensively studied, these three drugs can potentially become specific and effective anti-CPV drugs.

## 1. Introduction

Canine parvovirus (CPV), genus Protoparvovirus, a member of the family Parvoviridae (subfamily Parvovirinae), is a small, highly contagious, nonenveloped, single-stranded DNA virus [1]. CPV is a major causative agent of acute gastroenteritis, leukopenia and myocarditis in dogs, and typical clinical signs include vomiting, fever, and diarrhea. Generally, puppies aged 6 weeks to 6 months have been found to be more susceptible to CPV infection [2,3].

The CPV genome is approximately 5.2 kb in length, and contains two open reading frames (ORF), which encode 2 nonstructural proteins (NS1 and NS2) and two structural proteins (VP1 and VP2) [4,5,6]. After the emergence of CPV-2 in the late 1970s, CPV-2 and its variants have been reported in several countries and distributed among five continents [7].

Hoelzer et al. (2008) and Shackelton et al. (2005) previously reported that the CPV genomic substitution rate was similar to that of RNA viruses, and the earliest type of CPV-2 was replaced by three main subspecies—CPV-2a, CPV-2b, and CPV-2c—in just a few years [4,8,9,10]. Despite the widespread use of inactivated or live attenuated vaccines, the emergence of antigenic variants and the influence of maternal antibodies have contributed to questioning the efficacy of commercial vaccines [6,7]. In addition, there are no suitable antiviral drugs available for specific treatment of CPV infections. Therefore, the only treatment options for the infection are supportive and symptom-based care [11,12,13]. Thus, it is important to obtain antiviral drugs for potential therapeutic use in canine parvoviral diarrhea.

Drug repurposing screens, especially screening of existing drugs, was viewed as an alternative and efficient method of speeding up drug development [14]. Therefore, a Food and Drug Administration (FDA)-approved drug library has recently been widely used for successfully screening inhibitors against both DNA and RNA viruses, including Ebola (EBOV) [15,16], Zika (ZIKV) [14,17,18], and Hepatitis B (HBV) [19] viruses.

In this study, a high-throughput screening assay based on cytopathic effects (CPE) was used to screen an FDA-approved drug library for potential drugs capable of inhibiting CPV infection. One New CPV-2a variant SD6 was first used as the prototype for screening the library, then two other variants—BJ-1 (New CPV-2a), and SD3 (New CPV-2b)—were further used to evaluate the broad-spectrum antiviral activity of the identified drugs. The three identified inhibitors are promising drug candidates against CPV infection.

## 2. Materials and Methods

### 2.1. Cell and Viruses

Feline kidney fibroblast-like monolayer cell line (F81) was originally obtained from the American Type Culture Collection (ATCC). The cells were cultured in growth medium (GM), which consisted of Dulbecco’s Modified Eagle’s Medium (DMEM) (Gibco, Grand Island, NY, USA), supplemented with 10% heat-inactivated fetal calf serum, 100 U/mL penicillin, and 100 μg/mL streptomycin; while maintenance medium (MM) consisted of DMEM (Gibco), supplemented with 2% heat-inactivated fetal calf serum when used. New CPV-2a strain SD6 (297Ala, 426Asn), New CPV-2b strain SD3 (297Ala, 426Asp) and New CPV-2a strain BJ-1 (297Ala, 426Asn) were isolated and identified in our laboratory. The sequences encoding VP2 protein of SD6, SD3, and BJ-1 were deposited in GenBank under accession numbers MN101724, MN101725, and MN101726, respectively. These submitted sequences of SD6, SD3, and BJ-1 shared as high as 99.3%, 99.3%, and 99.1% nucleotide sequence identity with BR8-90 (DQ340411, New CPV-2a), CPV-2b/598/1995 (KF373568, New CPV-2b) and BR8-90 (DQ340411, New CPV-2a), respectively.

### 2.2. Cytopathic Effect-Based Antiviral Inhibitors Screening Assay

The FDA-approved library was purchased from Selleck Chemicals (USA), which consists of 1430 drugs. Prior to use, 4 μL of each 10 mM drug was transferred into 156 μL maintenance medium in a new 96-well plate for preparing a 250 μM stock solution. Firstly, 86 μL of F81 cells (2.5 × 10^4^ cells/well) were pretreated with 4 μL of each stock solution to obtain a final concentration of 10 μM for 1 h, then the drug-treated F81 cells were infected with 10 μL CPV at a multiplicity of infection (MOI) of 0.076. Normal and the CPV SD6-infected F81 cells containing a final concentration of 0.1% DMSO were used as positive and negative control, respectively. Cell viability was examined by TransDetect^®^ Cell Counting Kit (TransGen Biotech, Beijing, China) at 40 h postinfection. Optical density (OD) at 450 nm wavelength was collected using a SYNERGY H1 microplate reader (BioTek Instruments Inc., Winooski, VT, USA). OD_450_ values of wells without CPV infection served as a positive (cell) control indicating 100% CPE inhibition, and OD_450_ values of wells with SD6 infection served as a negative (virus) control indicating 0% CPE inhibition [14]. The percentage inhibition was calculated using the formula: percentage CPE inhibition = (OD_450_ of drug treated cells − OD_450_ of negative control)/(OD_450_ of positive control − OD_450_ of negative control) × 100 [20,21]. All drug plates were set-up in duplicates for primary screening, and drugs show greater than 20% CPE inhibition from the primary screen were used for second round of screening. The further validation of identified drugs was conducted in triplicates, and drugs show greater than 50% CPE inhibition were used for further analysis.

### 2.3. 50% Cytotoxicity Concentrations (CC_50_s) and 50% Antiviral Efficacy Concentrations (EC_50_s) Assays

Dose response experiments were performed to test CC_50_s and EC_50_s of drugs as described above with minor modifications. For CC_50_ assays, 96 μL of F81 cells (2.5 × 10^4^ cells/well) was mixed with 4 μL prediluted drugs at final concentrations ranging from 0.0024–160 μM. For EC_50_ assays, 86 μL of F81 cells (2.5 × 10^4^ cells/well) was pretreated with 4 μL prediluted drugs at final concentrations ranging from 0.0024–160 μM for 1 h, and then treated cells were infected with 10 μL CPV at an MOI of 0.076. Both CC_50_ and EC_50_ assays were done in triplicate. Cell cytotoxicity and inhibition of CPV infection were both examined after 40 h incubation using the TransDetect^®^ Cell Counting Kit (TransGen Biotech, Beijing, China). The CC_50_ and EC_50_ values were calculated via a best-fit Log (dose)-response curve-fitting in GraphPad Prism software (version 7.00, La Jolla, CA, USA) [21].

### 2.4. Absolute Quantitative PCR

Absolute quantification PCR was used to evaluate the anti-CPV effect. Total DNA was isolated from SD6 infected F81 cells using QIAamp DNA Mini Kit (Qiagen, Hilden, Germany) according to the manufacture’s instruction. CPV specific primers VP2-F 5′-CAAATAGAGCATTGGGCTTACC-3’ and VP2-R 5’-TCCCATTTGAGTTACACCACG-3’ were used to amplify the 119-bp fragment. The obtained fragment was cloned into pMD^®^18-T (Takara, Shiga, Japan), and the resulting positive clone was named pMD-VP2S for further use. To evaluate the antiviral effects of the three drugs of Nitazoxanide, Closantel Sodium and Closantel, F81 cells were seeded in 6-well plates at 7.5 × 10^5^ cells per well and pretreated with the 3 drugs at a final concentrations of 5 μM, 10 μM, and 20 μM, respectively, for 1 h, then treated cells were infected with CPV at MOI of 0.076 as described above. Cells treated with 0.1% DMSO were used as control. After 40 h incubation, total DNA was extracted from whole cell lysates by QIAamp DNA Mini Kit (Qiagen, Hilden, Germany). The quantitative standard curve was generated via quantitative real-time PCR of the plasmid pMD-VP2S preparations at serial dilutions of 10^6^, 10^5^, 10^4^, 10^3^, 10^2^, and 10 copies/μL. Each 20 μL qPCR reaction mixture contained 1 μL 10-fold diluted sample, 10 μL SuperReal PreMix Plus (SYBR Green) (TianGen Biotech, Beijing, China), and 0.2 μM of specific primers. All mixtures were then loaded into a StepOne Plus qPCR machine (Applied Biosystems, USA). The qPCR procedure was of 3 min at 95 °C, followed by 45 cycles of 5 s at 95 °C, and 30 s at 60 °C. The absolute number of DNA copies of the VP2 gene in the cells was determined according to the generated standard curve.

### 2.5. Immunofluorescence Aassay

Immunofluorescence assay (IFA) was used to further evaluate the antiviral effects of identified drugs. F81 cells in 96-well plates (2.5 × 10^4^ cells/well) were pretreated with 4 μL prediluted Nitazoxanide, Closantel Sodium, and Closantel at final concentrations of 5 μM, 10 μM, and 20 μM, respectively, for 1 h, then treated cells were infected with 10 μL CPV at MOI of 0.076. After 30 h postinfection, cells were fixed with 80% acetone, and then incubated with a 1:100 dilution of mouse anti-VP2 monoclonal antibody (INGENASA, Madrid, Spain) for 40 min, followed by incubation with a 1:200 dilution of fluorescein isothiocyanate-conjugated Goat anti-Mouse IgG (H+L) Highly Cross-Adsorbed Secondary Antibody (Invitrogen, Carlsbad, CA, USA). Finally, the cells were stained with 4′,6-diamidino-2-phenylindole (DAPI) in order to label cell nuclei in focus. After washing, the cells were examined with High Content imaging System (Operetta, PerkinElmer, Waltham, MA, USA) at 20× magnification.

### 2.6. Time of Addition Study

The time of addition experiment was used to test the drug inhibition stage of the CPV replication life cycle. Meanwhile, the inhibitory effects of the identified drugs at different time points following the addition of the three drugs after virus infection were also evaluated by this assay. Briefly, F81 cells were seeded in 96-well plates (2.5 × 10^4^ cells/well) and then infected with CPV (MOI = 0.076). Nitazoxanide, Closantel Sodium, Closantel, or 0.1% DMSO were added at −1 h (1 h pre-infection), 0 h (CPV infection), 0.5 h, 1 h, 2 h, 3 h or 6 h (post-infection) to determine the inhibitory effects at different time points of drug addition [20,21,22]. Cells treated with 0.1% DMSO served as control, and the effect on drug inhibition was evaluated using TransDetect^®^ Cell Counting Kit (TransGen Biotech, Beijing, China) at 40 h postinfection, as described above.

### 2.7. Immunoblotting

Western blot was also used to evaluate the antiviral activity of Nitazoxanide, Closantel Sodium, and Closantel against different subspecies of CPV variants. F81 cells were seeded in 6-well plates at 7.5 × 10^5^ cells per well and pretreated with the three drugs at final concentrations of 5 μM, 10 μM, and 20 μM, respectively, for 1 h. The treated cells were infected with CPV variants SD6 (New CPV-2a strain), SD3 (New CPV-2b strain) and BJ-1 (New CPV-2a strain), at an MOI of 0.076 as described above. Cells with 0.1% DMSO were used as control. After 40 h incubation, cells were harvested and lysed with ProteinExt^®^ Mammalian Total Protein Extraction Kit (TransGen Biotech, China). Equal amounts of cell lysates were analyzed via sodium dodecyl sulphate polyacrylamide gel electrophoresis (SDS-PAGE) and then transferred onto polyvinylidene difluoride (PVDF) membranes (Millipore, Burlington, MA, USA). After blocking with 5% milk-TBS-Tween 20 for 1 h at room temperature, anti-VP2 monoclonal antibody (1:800 dilution, INGENASA, Madrid, Spain), and beta-actin monoclonal antibody (AC-15) (1:4000 dilution, Thermo Scientific, Waltham, MA, USA) were added and incubated overnight at 4 °C, blots were further incubated with horseradish-peroxidase(HRP)-conjugated goat anti-mouse IgG for 1 h at 37 °C. The immunoreactive bands were detected using a SuperSignal™ West Pico PLUS Chemiluminescent Substrate Kit (Thermo Scientific, USA) and imaged using a chemiluminescence apparatus (Proteinsimple, USA). Band intensities were measured using the Image J software, and viral VP2 protein expression was first compared with beta Actin expression, and then normalized to the 0.1% DMSO-treated group.

### 2.8. Caspase-3 Assay

F81 cells were seeded in 96-well plates (2.5 × 10^4^ cells/well) and pretreated with 4 μL Nitazoxanide, Closantel Sodium, and Closantel with final concentrations of 10 μM, then infected with CPV (MOI = 0.076). Cells were incubated for 4 h, 8 h, 12 h and 24 h at 37 °C and 5% CO_2_. The assay was performed in triplicates. Caspase-Glo 3/7 assay kit (Promega, USA) was used to detect pro- or antiapoptotic effects of the identified drugs [14,23]. The luminescence of each sample was measured using a SYNERGY H1 microplate reader (BioTek Instruments Inc., Winooski, USA) according to the manufacturer’s instructions [14].

### 2.9. Statistical Analysis

The CC_50_s and EC_50_s of drugs were determined by a best-fit Log(dose)-response curve-fitting in GraphPad Prism 7. One-way analysis of variance (ANOVA) and Dunnett’s multiple comparisons test were used to analyze data. Statistical significances are denoted as follows; * *p* < 0.05; ** *p* < 0.01; *** *p* < 0.005; **** *p* < 0.001.

## 3. Results

### 3.1. Screening Drug Inhibitors against CPV Infection in F81 Ccells

**Figure 1:**
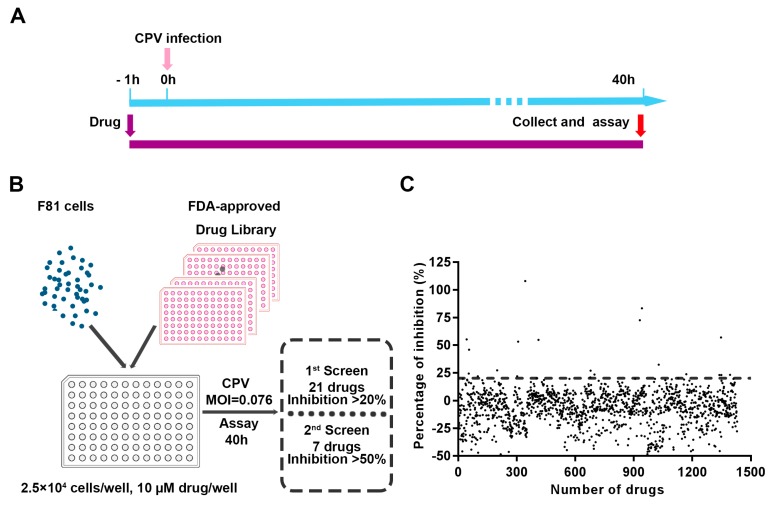
Screening of the FDA-approved compound library for inhibitors of CPV replication. (**A**) Experimental timeline of drug treatment and CPV infection. F81 cells were seeded in 96-well plates and pretreated with 10 μM drugs for 1 h before CPV infection, then cell viability was examined using the TransDetect^®^ Cell Counting Kit at 40 h postinfection. (**B**) Flow chart of drug screen using CPE-based assay. Briefly, F81 cells per well were pretreated with 10 μM drugs for 1 h, and then infected with 0.076 MOI CPV, cell viability was detected at 40 h postinfection as described above, antiviral inhibitors against CPV were determined according to the percentage CPE inhibition. Twenty-one drugs showing >20% CPE inhibition from the primary screen were used for a second round of screening, and seven drugs with percentage inhibition >50% were further identified. (**C**) Scatter plot of percentage CPE inhibition results for 1430 FDA-approved drugs, numbers in X axis mean the species of the tested drugs, each number corresponds to a specific drug, and the order is the same as that provided in the manual of the FDA-approved drug library, each dot shows the mean percentage CPE inhibition in the presence of 10 μM tested drug.

In this study, a CPE-based high-throughput screening assay was used to screen CPV inhibitors from an FDA-approved drug library. The timeline of drug treatment and CPV infection, as well as the flow chart of the CPE-based assay, are shown in Figure 1A,B. In the primary screen (First round), the Z’ factor was between 0.68 and 0.83 across all 17 drug plates. As the assay quality control index Z’ factors were >0.5 in all plates, it demonstrated that the CPE-based screening assay was suitable for screening anti-CPV drugs. The mean percentage CPE inhibition of each drug was plotted in Figure 1C.

**Figure 2:**
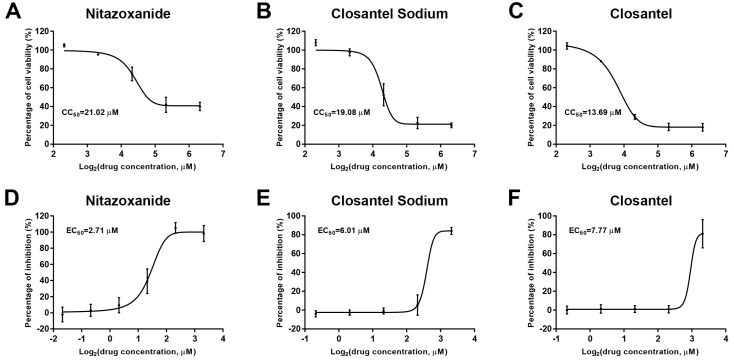
Evaluation of cytotoxicity and anti-CPV efficacy of three identified drugs. Dose-dependent curves show cell viability of F81 cells with 2-fold serial dilution concentrations of Nitazoxanide (**A**), Closantel Sodium (**B**), and Closantel (**C**). Dose-dependent curves show the anti-CPV efficacy of 2-fold serially diluted Nitazoxanide (**D**), Closantel Sodium (**E**), and Closantel (**F**). Error bars represent standard errors from three independent experiments. The CC_50_s and EC_50_s were determined by a best-fit Log(dose)-response curve-fitting in GraphPad Prism 7.

Twenty-one drugs with >20% CPE inhibitions, identified during the first round of screening, were used for the second round of screening. The drug name, catalogue number of Selleck, and the final percentage CPE inhibition of the 21 drugs are listed in Table S1, and the inhibitory effects of these drugs, when these drugs were added 1 h post-virus infection are also listed in Table S1. Seven drugs with percentage CPE inhibitions >50% were selected for further CC_50_ and EC_50_ assays, and the results are shown in Figure 2 and Figure S1 and are also listed in Table 1.

The top three drugs—Nitazoxanide, Closantel Sodium, and Closantel—were identified with higher percentage CPE inhibition of 106.59 ± 2.79, 69.76 ± 6.06, and 80.64 ± 7.87%, respectively, at 10 μM concentration (Table S1). Moreover, all three drugs showed a dose-dependent inhibition of CPV infection (Figure 2).

### 3.2. Validation of Anti-CPV Drug Candidates by qPCR and IFA

**Figure 3:**
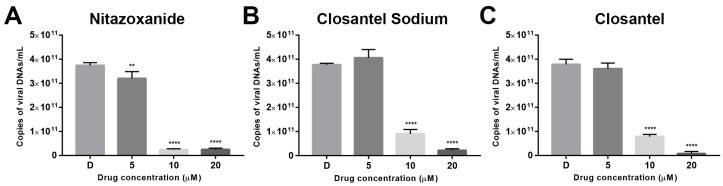
The antiviral effects of the identified drugs were analyzed by quantification of viral DNAs. F81 cells seeded in 6-well plates were pretreated with 5 μM, 10 μM, or 20 μM of Nitazoxanide (**A**), Closantel Sodium (**B**), or Closantel (**C**) before CPV infection. Total DNA was extracted at 40 hpi from whole cell lysates. The absolute number of copies of the VP2 gene in the cells was determined according to the generated standard curve. Error bars represent standard errors from three independent experiments. Statistical analysis was normalized to Control (0.1% DMSO-treated cells, marked as D in the figure) and carried out using one-way ANOVA and Dunnett’s multiple comparisons test. * *p* < 0.05; ** *p* < 0.01; *** *p* < 0.005; **** *p* < 0.001.

From the absolute qPCR results, dose-dependent reduction in the copy numbers of CPV viral DNA were observed with increasing concentrations of Nitazoxanide (Figure 3A), Closantel Sodium, (Figure 3B) or Closantel (Figure 3C). When CPV-infected F81 cells were treated with the three drugs at 10 μM, the CPV viral DNA copy numbers of 1mL whole cell lysates significantly reduced to 0.07% (Nitazoxanide), 24.04% (Closantel Sodium), and 20.83% (Closantel) compared with the 0.1% DMSO-treated group (Figure 3).

**Figure 4:**
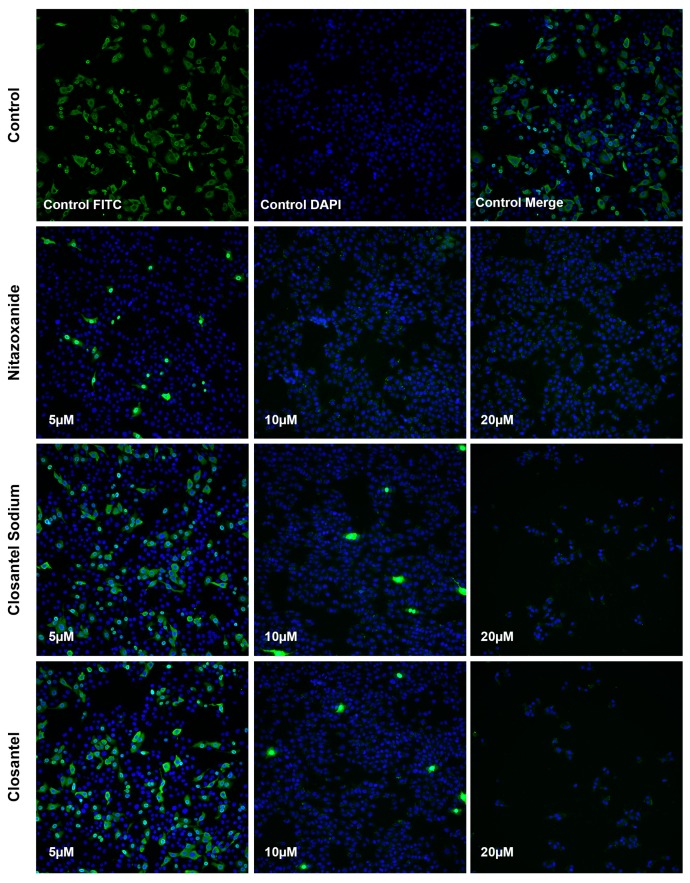
The antiviral effects of identified drugs were analysed via IFA. F81 cells were treated with 0.1% DMSO (Control) or 5 μM, 10 μM, or 20 μM of Nitazoxanide, Closantel Sodium, or Closantel before CPV infection. Then cells were fixed with 80% acetone at about 30 hpi and immunostained for VP2 protein (green), while nuclei (blue) were stained with DAPI.

As shown in Figure 4, CPV infection could be inhibited in the presence of Nitazoxanide, Closantel Sodium, and Closantel at a concentration of 5 μM. Few cells were CPV-positive when treated with 10 μM of the drugs. Almost no green signals were detected in all F81 cells treated with 20 μM of the drugs. The results confirmed that these identified drugs inhibited CPV infection in a dose-dependent manner, which was consistent with the qPCR assay results.

### 3.3. Inhibitory Effects of Anti-CPV Drugs at Different Time Points.

**Figure 5:**
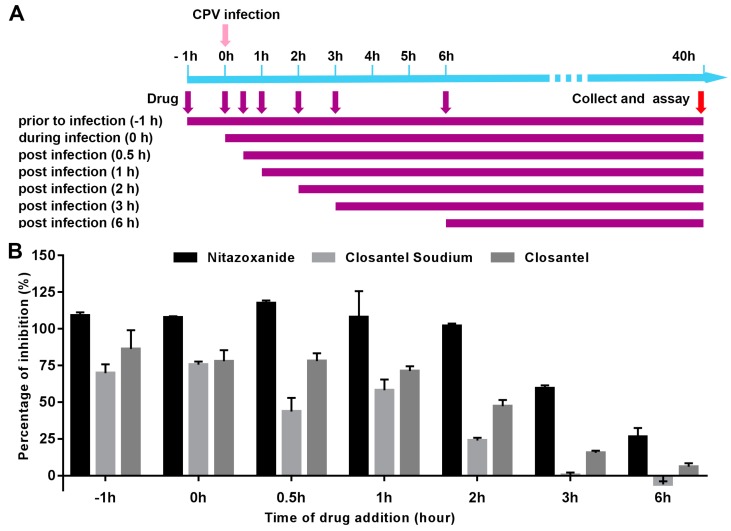
Time of addition analysis of the antiviral effects of identified drugs. (**A**) Timeline of time of addition assay. F81 cells were inoculated with CPV and treated with 10 μM Nitazoxanide, Closantel Sodium, and Closantel, respectively, at different time points, designated prior to infection (−1 h), during infection (0 h), postinfection (0.5 h, 1 h, 2 h, 3 h, or 6 h). Antiviral effects were determined according to the percentage CPE inhibition. (**B**) Percentage CPE inhibition of Nitazoxanide, Closantel Sodium, and Closantel added at different time points, presented in bar graphs. Error bars represent standard errors from three independent experiments.

Consistent with screening results, all three drugs showed anti-CPV effects when added 1 h before virus infection (pre-infection). The inhibitory effects of Nitazoxanide, Closantel Sodium, and Closantel were 108.00 ± 17.74, 58.14 ± 7.28, and 71.28 ± 3.17, respectively, when the three drugs were added 1 h post-virus infection (Figure 5). The drugs inhibited the early processes of the CPV replication cycle, and the inhibition effects were relatively high within 2 h postinfection (Figure 5). In addition, the anti-CPV activity of Nitazoxanide was observed when the drug was added 3 h postinfection, suggesting that Nitazoxanide may partially have the ability to inhibit CPV infection at the stage of viral replication.

### 3.4. Inhibitory Effects of Anti-CPV Drugs on Different CPV Variants

**Figure 6:**
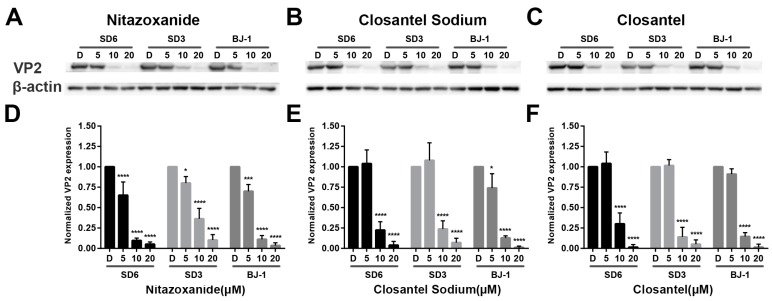
Potential broad-spectrum anti-CPV activity of identified drugs. F81 cells were seeded in 6-well plates and pretreated with 5 μM, 10 μM, or 20 μM of Nitazoxanide (**A**), Closantel Sodium (**B**), or Closantel (**C**) for 1 h, respectively. Then treated cells were infected with various CPV strains SD6, SD3 or BJ-1. Cells were harvested and lysed at 40 hpi for western blot analysis. Band intensities were then measured using software Image J, and VP2 expression was analyzed and compared to beta Actin expression. Relative expression levels for Nitazoxanide- (**D**), Closantel Sodium- (**E**), and Closantel (**F**)-treated results are presented in bar graphs. Error bars represent standard errors from three independent experiments. Statistical analysis was compared to Control (0.1% DMSO-treated cells, marked as D in the figure) and carried out using one-way ANOVA and Dunnett’s multiple comparisons test. * *p* < 0.05; ** *p* < 0.01; *** *p* < 0.005; **** *p* < 0.001.

Western blot was also used to evaluate the broad-spectrum antiviral activity of identified drugs against different subspecies of three CPV variants. Dose-dependent reductions in VP2 expression and the quantification of relative expression levels are shown in F81 cells treated with Nitazoxanide (Figure 6A,D), Closantel Sodium (Figure 6B,E) or Closantel (Figure 6C,F), respectively. Nitazoxanide treated at 10 μM reduced relative expression of VP2 in different CPV variants to 9.68% (SD6), 36.29% (SD3), and 11.22% (BJ-1), respectively (Figure 6D). Closantel Sodium treated at 10 μM reduced relative expression of VP2 in different CPV variants to 22.50% (SD6), 23.85% (SD3) and 12.83% (BJ-1), respectively (Figure 6E). Closantel treated at 10 μM reduced relative expression of VP2 in different CPV variants to 30.1% (SD6), 10.78% (SD3), and 14.58% (BJ-1), respectively (Figure 6F). All the identified drugs showed inhibitory ability against CPV variants SD6, SD3, and BJ-1.

### 3.5. Nitazoxanide-Induced Apoptosis Involved in Antiviral Effect.

**Figure 7:**
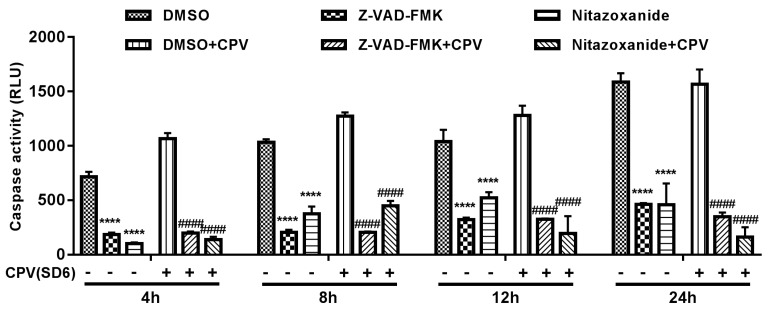
Caspase 3/7 activity of Nitazoxanide treated F81 cells. F81 cells were pretreated with 0.1% DMSO, Z-VAD-FMK (as a control to inhibit apoptosis), or Nitazoxanide for 1 h, then Caspase 3/7 activity of F81 cells with or without CPV infection were analyzed at 4 hpi, 8 hpi, 12 hpi, and 24 hpi. Statistical analysis was carried out using one-way ANOVA and Dunnett’s multiple comparisons test. * *p* < 0.05; ** *p* < 0.01; *** *p* < 0.005; **** *p* < 0.001 (compared to 0.1% DMSO-treated cells without CPV infection, at each time point). # *p* < 0.05; ## *p* < 0.01; ### *p* < 0.005; #### *p* < 0.001 (compared to 0.1% DMSO-treated cells with CPV infection, at each time point).

Doley et al. (2014) reported that CPV could induce caspase-dependent (involving extrinsic, intrinsic, and endoplasmic reticulum pathways) apoptosis in Madin-Darby canine kidney (MDCK) cells [24]. In order to evaluate whether drug-associated apoptosis was involved in the antiviral effect, we measured the caspase-3 activity of drug-treated cells with or without CPV infection. As shown in Figure 7, the antiapoptotic effects were observed within 12 h in Nitazoxanide-treated F81 cells with or without CPV infection. Therefore, Nitazoxanide-associated caspase activation reduction (apoptosis) might be involved in the antiviral effect of the drug. Closantel Sodium- or Closantel-treated cells had no pro- or antiapoptotic effects (Figure S2); hence, the antiviral effects of these two drugs may not be due to drug-associated apoptosis.

## 4. Discussion

CPV is a widely distributed virus and contains at least three main subspecies: CPV-2a, CPV-2b, and CPV-2c [4,6,8]. Currently, commercial vaccines cannot provide complete protection against all CPV variants. Moreover, no effective drug is available to control CPV infection except for supportive and symptom-based care. Hence, it is important to develop an alternative treatment against CPV infection. In this study, we developed a CPE-based assay to screen CPV inhibitors from a FDA-approved drug library, and successfully identified three FDA-approved CPV inhibitors. These drugs might provide potential treatment options for anti-CPV infections.

Although the selectivity index (SI) of Gemcitabine HCl, Cladribine, Gemcitabine, and Trifluridine were at a higher level (Table 1), the percentage CPE inhibition of the four drugs was always maintained at a lower level (Figure S1). The maximum percentage CPE inhibitions for the drugs were between 51.80 ± 2.48 and 68.37 ± 7.79, which are relatively lower CPE inhibition levels that would not increase with increased drug concentration. Therefore, Nitazoxanide, Closantel Sodium, and Closantel were selected for further study.

As mentioned above, the identified drugs Nitazoxanide, Closantel Sodium, and Closantel can reduce the copy numbers of CPV viral DNA to 0.07%, 24.04%, and 20.83%, respectively, compared with the 0.1% DMSO-treated control (Figure 3). Meanwhile, the IFA result also showed that these identified drugs inhibited CPV infection in a dose-dependent reduction manner. Western blot showed that 10 μM Nitazoxanide treatment reduced relative VP2 expression in three CPV variants SD6, SD3, and BJ-1 to 9.68%–36.29%, and the reduction rates following 10 μM Closantel Sodium and Closantel treatment were 12.83%–23.85% and 10.78%–30.1%, respectively (Figure 6). These results indicated that the identified drugs had significant inhibitory effects against CPV infection in F81 cells. 

In previous studies, two drugs Oseltamivir and Cidofovir were added in the second round of screening. As a neuraminidase (NA) inhibitor, Oseltamivir has been used to treat the human influenza virus. Savigny and Macintire (2010) used Oseltamivir for CPV enteritis and found that the Oseltamivir-treated group gained a significant increase of weight and had no changes in white blood cell (WBC) count compared to the control group; however, the authors also reported that no obvious advantage had been established [25]. Cidofovir is a broad-spectrum anti-DNA virus drug, which had been evaluated for the treatment of human papillomavirus (HPV)-associated tumors [26]. Our CPE-based screening assay showed that the percentage CPE inhibition of Oseltamivir and Cidofovir were 2.13 ± 2.41 and −1.28 ± 1.03 (Table S1), respectively, and that these two drugs had no anti-CPV effects on F81 cells.

Previously, Nitazoxanide was used to treat cryptosporidiosis, giardiasis, and other parasitic infections [27]. Recently Nitazoxanide was reported to inhibit various DNA and RNA viruses, including hepatitis B virus (HBV) [28,29], human cytomegalovirus (HCMV) [30], influenza A virus [31], hepatitis C virus [32], norovirus [33], rotavirus [34], Japanese encephalitis virus (JEV) [35], coronavirus [36] chikungunya virus (CHIKV) [20], human immunodeficiency virus (HIV) [37], and ZIKV [38]. 

The antiviral mechanism of Nitazoxanide remains unclear for now. Nitazoxanide could impair the terminal glycosylation of the influenza A hemagglutinin protein or the formation of E1-E2 (Rubella virus surface glycoproteins) complex of the Rubella virus (RV), thus affecting the assembly of influenza A virus and RV, respectively [31,39]. In addition, Nitazoxanide could also hinder the interactions between the proteins NSP5 and NSP2 of Rotavirus or the interactions between proteins NS2B and NS3 of ZIKV and dengue virus 2 (DENV2) [34,40]. Mercorelli et al. (2016) also reported that Nitazoxanide can inhibit the transcriptional activation properties of the HCMV immediate-early 2 (IE2) protein [30]. These results indicated the virus-specific effects of Nitazoxanide.

Since Nitazoxanide can inhibit the replication of various DNA and RNA viruses, various studies have focused on identifying host factors to explain the broad antiviral activities of Nitazoxanide. Ashiru et al. (2014) reported that Nitazoxanide depleted intracellular Ca^2+^ stores, besides the phosphorylation of PKR and eIF2α, further affecting N-linked glycosylation of the bovine viral diarrhea virus (BVDV) E2 protein and trafficking from the ER to the Golgi [41]. Nitazoxanide can elicit antiviral innate immunity and reduce the HIV replication by activating the interferon system and further expression of various interferon-stimulated genes (ISGs) [37]. In addition, Nitazoxanide might block the production of acetyl-CoA, which is a required metabolic intermediates for Vaccinia virus (VACV) reproduction. In general, further studies are still required to clearly elucidate the antiviral mechanism of Nitazoxanide [27].

Closantel sodium and Closantel were also identified and shown to have anti-CPV activities. Closantel is a salicylanilide derivative and is considered to be an anthelmintic agent in livestock [42]. The antiangiogenesis and anticancer effects of Closantel sodium and Closantel have also been previously reported [43]. However, to our knowledge, neither the antiviral activity nor the antiviral mechanisms of Closantel sodium and Closantel have been reported before. Previous studies have shown that Closantel inhibited B-Raf (a serine/threonine kinase) V600E [44], adenine nucleotide translocase (ANT) [45], SPAK and OSR1 kinase [46]. In addition, Senkowski et al. (2015) reported that Closantel could inhibit mitochondrial respiration as well [47]. These reports may contribute to further studies on the antiviral mechanism of Closantel.

## 5. Conclusions

In this study, which is aimed at identifying anti-CPV drugs for potential therapeutic use, a CPE-based assay was developed for screening CPV inhibitors from an FDA-approved drug library. After screening, the top three drugs, Nitazoxanide, Closantel Sodium, and Closantel, with higher percentage CPE inhibition, were selected and further confirmed by qPCR and IFA. In addition, the identified drugs can inhibit different subspecies of CPV variants and displayed broad-spectrum antiviral activity against CPV. Hence, these drugs may provide potential options for the treatment of CPV infection.

## Figures and Tables

**Table 1:** 50% cytotoxicity concentration (CC_50_), 50% antiviral efficacy concentration (EC_50_), and selectivity index (SI) of identified anti-CPV drugs.

Hit drugs	CC_50_ (µM)	EC_50_ (µM)	SI
Nitazoxanide	21.02	2.71	7.76
Closantel Sodium	19.08	6.01	3.17
Closantel	13.69	7.77	1.76
Gemcitabine HCl	141.6	0.68	208.24
Cladribine	40.21	0.32	125.66
Gemcitabine	40.03	0.62	64.56
Trifluridine	>160	9.35	>17.11

## Supplementary Materials

The following are available online at https://www.mdpi.com/1999-4915/11/8/742/s1, Table S1: Percentage inhibition (%) of 21 drugs and 2 other tested drugs, Figure S1: Evaluation of cytotoxicity and anti-CPV efficacy of other four drugs, Figure S2: Caspase 3/7 activity of Closantel Sodium or Closantel treated F81 cells.
