# Supplementary material for: Inhibitory Effects of Antiviral Drug Candidates on Canine Parvovirus in F81 cells
Source: Viruses. 2019 Aug 13;11(8):742. doi: 10.3390/v11080742 (PMC6724046; doi:10.3390/v11080742)
Supplement: Supplementary file 1 [file viruses-11-00742-s001.pdf]

Table S1. Percentage inhibition (%) of 21 drugs and 2 other tested drugs

	Plate number	Well number	Catalogue number	Drug name	Percentage inhibition (mean ± s.d.%)[a]	Percentage inhibition (mean ± s.d.%)[b]
**1**	4	H2	S1627	Nitazoxanide	106.59 ± 2.79	108.00 ± 17.74
**2**	11	F7	S4106	Closantel	80.64 ± 7.87	71.28 ± 3.17
**3**	11	E7	S4105	Closantel Sodium	69.76 ± 6.06	58.14 ± 7.28
**4**	16	D6	S1149	Gemcitabine HCl	64.18 ± 0.97	48.93 ± 3.57
**5**	5	F4	S1778	Trifluridine	57.11 ± 5.45	62.4 ± 6.3
**6**	4	D10	S1714	Gemcitabine	56.1 ± 2.09	42.8 ± 6.13
**7**	1	D10	S1199	Cladribine	50.92 ± 1.58	46.33 ± 2.01
**8**	1	E11	S1210	Methotrexate	43.67 ± 6.07	41.28 ± 1.95
**9**	1	E9	S1192	Raltitrexed	35.11 ± 3.19	42.1 ± 4.45
**10**	8	H5	S2517	Maprotiline HCl	34.2 ± 7.09	30.62 ± 3.14
**11**	14	C3	S4591	Nitroxoline	32.28 ± 7.83	11.77 ± 7.45
**12**	12	F5	S4218	Amoxapine	30.06 ± 6.89	34.69 ± 9.61
**13**	2	B1	S1218	Clofarabine	29.54 ± 3.8	34.86 ± 5.45
**14**	3	C1	S1394	Pizotifen Malate	29.2 ± 2.72	26.48 ± 2.91
**15**	16	D8	S1648	Cytarabine	28.66 ± 4.67	30.5 ± 4.22
**16**	8	F7	S2541	Clomipramine HCl	26.72 ± 8.8	24.01 ± 8.42
**17**	17	B7	S7785	Pemetrexed Disodium Hydrate	26.56 ± 2.12	30.28 ± 5.74
**18**	16	C6	S1135	Pemetrexed	26.53 ± 4.13	39.29 ± 10.64
**19**	4	D5	S1652	Monobenzone	18.47 ± 1.15	17.98 ± 4.7
**20**	8	F6	S2528	Ciclopirox	14.12 ± 5.32	9.76 ± 4.58
**21**	13	G6	S4416	Trimipramine Maleate	12.85 ± 3.68	20.2 ± 3.13
**22**			S2597	Oseltamivir	2.13 ± 2.41	3.6 ± 2.89
**23**			S1516	Cidofovir	-1.28 ± 1.03	-6.1 ± 4.71

[a] The percentage CPE inhibition of drugs were obtained by treating cells with drugs 1 hour prior to virus infection.

[b] The percentage CPE inhibition of drugs were obtained by adding drugs 1 hour post virus infection.

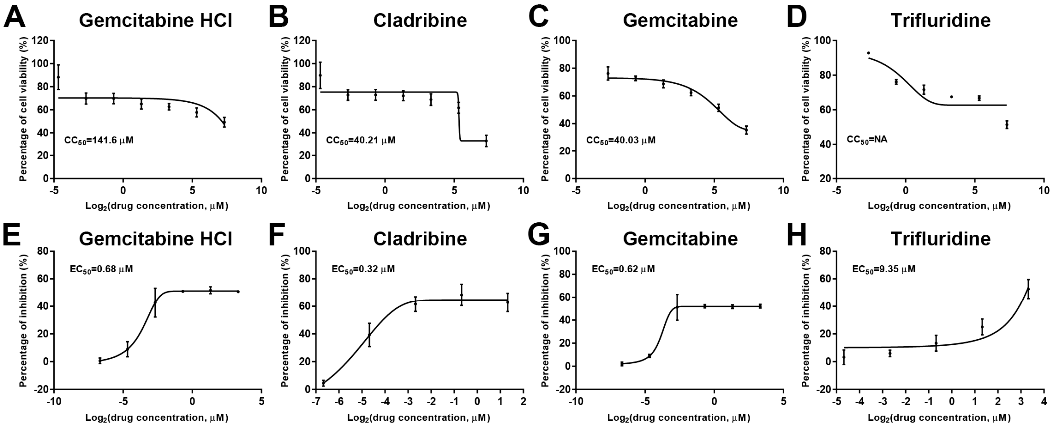

Figure S1. Evaluation of cytotoxicity and anti-CPV efficacy of other four drugs. Dose-dependent curves show cell viability of F81 cells with 4-fold serial dilution concentrations of Gemcitabine HCl (A), Cladribine (B), Gemcitabine (C) and Trifluridine (D) Dose-dependent curves show the anti-CPV efficacy of 4-fold serially diluted Gemcitabine HCl (E), Cladribine (F), Gemcitabine (G) and Trifluridine (H). Error bars represent standard errors from three independent experiments. The CC_{50}s and EC_{50}s were determined by a best-fit Log(dose)-response curve-fitting in GraphPad Prism 7.

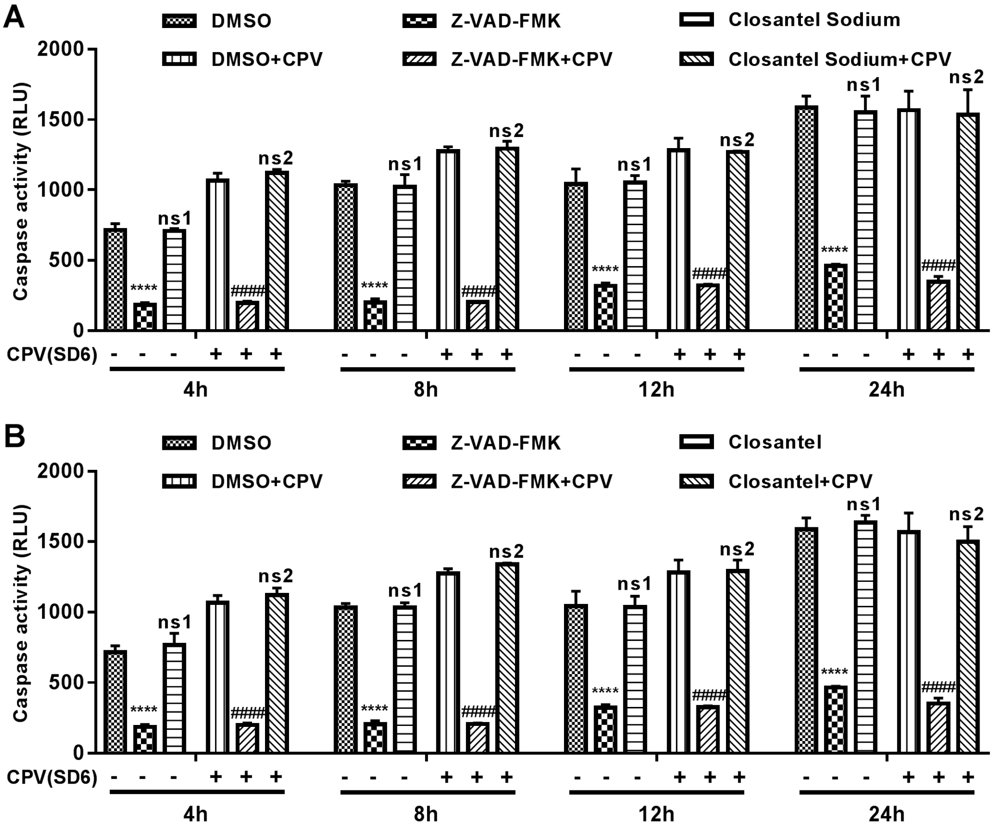

Figure S2. Caspase 3/7 activity of Closantel Sodium or Closantel treated F81 cells. F81 cells were pre-treated with 0.1% DMSO, Z-VAD-FMK (as a control to inhibit apoptosis), or Closantel Sodium (A) or Closantel (B) for 1 h, then Caspase 3/7 activity of F81 cells with or without CPV infection were analysed at 4 hpi, 8 hpi, 12 hpi and 24 hpi. Statistical analysis was carried out using one-way ANOVA and Dunnett's multiple comparisons test. ns1; * $p < 0.05$; ** $p < 0.01$; *** $p < 0.005$; **** $p < 0.001$ (compared to 0.1% DMSO-treated cells without CPV infection, at each time point). ns2; # $p < 0.05$; ## $p < 0.01$; ### $p < 0.005$; #### $p < 0.001$ (compared to 0.1% DMSO-treated cells with CPV infection, at each time point).
